# Effect of aspirin on PET parameters in primary non-small cell lung cancer and its relationship with prognosis

**DOI:** 10.1186/s12885-020-06983-2

**Published:** 2020-06-03

**Authors:** Jinghua Chen, Junxian Xia, Jiacheng Huang, Ruilian Xu

**Affiliations:** 1 First Affiliated Hospital of Southern University of Science and Technology, Shenzhen, 518020 P. R. China; 2 Second Clinical Medicine College, Jinan University, Shenzhen, 518020 P. R. China; 3 Department of Medical Oncology, Shenzhen People’s Hospital, Shenzhen, 518020 P. R. China

**Keywords:** Aspirin, SUVmax, 18 F-FDG, Non-small cell lung cancer, Prognosis

## Abstract

**Background:**

18 F-FDG is a glucose analogue whose metabolic index SUV can effectively reflect the metabolic level of tumor microenvironment. Aspirin can affect the uptake of 18F-FDG by cancer cells, reducing the SUVmax value of primary tumors, exerting antitumor effect. This study aimed to evaluate the prognostic value of long-term aspirin and the relationship between aspirin intake and PET parameters value of primary tumor in non-small cell lung cancer (NSCLC).

**Methods:**

Eighty-one NSCLC patients were recruited and divided into two groups: aspirin medication group and control group, who underwent surgery and had pathological diagnosis data between January 2012 and December 2016. Clinical characteristics were retrospective analyzed to evaluate the possibility of clinical prognosis, respectively. Kaplan-Meier curves and a Cox proportional hazard model were applied to evaluate the predictors of prognosis.

**Results:**

The PET/CT SUVmax of the primary tumor in the aspirin group was lower than that in the control group (*P* < 0.05). Compared with the control group, the SUVmax, SUVmean and TLG of the primary tumor in aspirin group were lower, but the MTV value had no significant difference. Cox regression analysis showed that N stage and TNM stage were predictors of the prognosis. There was a significant difference in the use of aspirin in NSCLC patients.

**Conclusion:**

Aspirin can reduce SUVmax, SUVmean and TLG in primary tumor and aspirin can improve the prognosis of patients with NSCLC.

## Background

Aspirin is the most used, longest-lived, non-steroidal anti-inflammatory drug in the world. Epidemiological and clinical experiments have shown that aspirin can prevent and treat various tumors such as lung cancer, colorectal cancer, cholangiocarcinoma, esophageal cancer and gastric cancer, in addition to antipyretic and anti-platelet aggregation [1–5]. A meta-analysis of randomized studies including five assessments of daily aspirin for prevention of cardiovascular events showed aspirin reduced the risk of 35% of fatal adenocarcinomas and 31% of metastatic adenocarcinomas, and reduced the risk of 74% of current and follow-up transitions [6, 7]. However, some studies have concluded that aspirin does not reduce the risk of cancer [8–10].

18 F-deoxyglucose (FDG) positron emission tomography/computed tomography (PET/CT) effectively combines two images of anatomical structure and metabolic function [11]. As a non-invasive method, it is widely used in clinical staging and efficacy evaluation. 18 F-FDG is a glucose analogue whose metabolic index of maximum standardized uptake value (SUVmax) can effectively reflect the metabolic level of tumor microenvironment. The existing studies have suggested that the SUVmax of primary lung cancer is associated with clinical stage, pathological type, lymph node status, and tumor proliferation rate [12, 13]. Other quantitative parameters of PET / CT can represent the metabolic load of tumor, such as the metabolic volume (MTV) and the total glycolysis (TLG), which also has certain prognostic value. High SUVmax has become one of the poor prognostic factors in patients with NSCLC. Tracer 18F-FDG can be transported into cells mainly through glucose transporters protein (Glut). Animal studies have shown that aspirin can destroy the Glut of tumor cells, and the uptake of 18F-FDG in tumor cells is positive correlated with the expression of Glut [14]. In addition, previous studies have shown that long-term use of aspirin can reduce the SUVmax of primary colorectal cancer. The aim of this study is to investigate the prognostic value of long-term aspirin and the relationship between aspirin intake and SUVmax value of primary tumor on PET/CT SUVmax in primary non-small cell lung cancer (NSCLC), and to provide a reference for further development of treatment strategies and prognosis.

## Methods

### Patients and population

Eighty-one patients with non-small cell lung cancer patient data from January 2012 to December 2016 were selected.

Entry criteria:

1. Patients underwent baseline evaluation with PET/CT before surgery.
2. Clinical stage was from I to III.
3. No neoadjuvant radiotherapy or chemotherapy before surgery.
4. Completely record information about the patient’s use of aspirin, including usage, dosage, and time of use. Meanwhile, five patients were excluded for the early loss of follow-up. According to the application of aspirin or not, it was divided into aspirin medication group and control group. Patients in the aspirin medication group received oral aspirin 75 mg/day for at least 5 years. The control group did not take or occasionally take aspirin. All patients underwent regular follow-up after surgery (reviewed once every 3 months in 2 years and once every 6 to 8 months after 2 years). The recorded follow-up time was from 1 to 160 months after surgery.

### Whole body 18 F-FDG PET/CT examination

The scanning device was PHILIPS GEMINI TF64-PET/CT device. The scanning parameters were tube voltage 120KV, tube current 50 mA, layer thickness 5 mm, layer spacing 5 mm. All patients underwent PET/CT before blood glucose control, fasting for 6 h or more, and banned sugary drinks. The synthesized 18F-fluorodeoxyglucose (18F-FDG) was intravenously injected into the patient at a dose of 0.125 mCi/KG. The patients rested for 1 h after injecting the drug, and a whole body scan was performed after urination. The scan ranged from cranial crest to mid-femur. More than two experienced nuclear medicine physicians performed the attenuation correction and iterative reconstruction of the obtained image. With SUV ≥ 2.5 as the threshold, the region of interest (ROI) was set in the radionuclide concentration area. The SUVmax, SUVmean and MTV of the area were calculated by computer software, and TLG: TLG = MTV × SUVmean was calculated to obtain TLG of each focus.

### Statistical processing and follow-up

Analysis was performed by using SPSS 21.0 statistical software. The surgery day was the initial point of observation, and the metastatic or recurrence day was the end point of observation. This time period was called disease-free survival (DFS). The chi-square test was used for the count data, the t-test was used for the measurement data. The survival rate was calculated by the Kaplan-Meier method, the survival curve was plotted, and the log-rank test was performed. Multivariate survival analysis was performed using a Cox proportional hazards regression model with forward stepwise regression. All statistical analyses were statistically significant at *P* < 0.05.

## Results

### Comparison of clinical features between the two groups

Among the 81 cases included in the study, 60 were men and 21 were women, aged 32–71 years (median age 56 years). The pathological types of lung cancer included 32 cases of adenocarcinoma, 37 cases of squamous cell carcinoma, and 12 cases of large cell carcinoma. The level of differentiation was 18 cases with high differentiation, 28 cases with moderate differentiation, and 35 cases with poor differentiation. According to UICC 2017 lung cancer staging criteria, 14 cases of stage I, 58 cases of stage II, and 9 cases of stage III were included, all of which were postoperative staging. There were 23 patients (28.4%) in the aspirin group and 58 patients (71.6%) in the control group. At the end of the follow-up, 2 patients were lost to follow-up, 75 patients had tumor recurrence or metastasis, and 4 patients had no disease progression. The median follow-up time was 52 months and there were no significant differences in age, gender, pathological stage, pathological type, and tumor differentiation between the aspirin group and the control group (Table 1).

**Table 1:** Patient demographics and clinical characteristics

Characteristic	Aspirin group (*n* = 23)	Control group (*n* = 58)		*P* value
Age	54.1 ± 9.3	55.1 ± 9.6	t = 0.118	0.793
Gender				
Male	18	42	x^2^ = 0.293	0.405
Female	5	16		
Differentiation				
Well	5	13	z = −0.051	0.96
Moderate	8	20		
Poor	10	25		
Pathologic types				
Adenocarcinoma	9	23	x^2^ = 0.102	0.965
Squamous cell	11	26		
Large cel	3	9		
Stage			z = −0.695	0.487
I	2	12		
II	19	39		
III	2	7		
SUVmax	7.3 ± 3.3	12.1 ± 5.1	t = 4.147	0.005
Tumor size	3.4 ± 1.1	3.7 ± 1.1	t = 0.999	0.359
N classification			z = −0.57	0.569
0	14	40		
1	8	14		
2	1	4		
T classification			Z = -0.038	0.97
1	6	16		
2	16	38		
3	1	4		

### Effect of aspirin on PET / CT metabolic parameters in primary NSCLC

The PET/CT SUVmax, SUVmean and TLG of primary NSCLC in aspirin group were lower than those in control group, which indicated that long-term aspirin administration could reduce SUVmax, SUVmean and TLG of primary lung cancer (*p* < 0.05). There was no significant difference in MTV between aspirin group and control group (*p* > 0.05). (Table 2).

**Table 2:** Comparison of PET / CT metabolic parameters between aspirin group and control group of patients with primary lung cancer

	Aspirin group (*n* = 23)	Control group (*n* = 58)	*t*	*P*-value
SUVmax	7.3 ± 3.3	12.1 ± 5.1	4.147	0.005
SUVmean	6.1 ± 2.7	9.8 ± 3.8	4.303	0.004
MVT	17.21 ± 17.85	19.85 ± 18.70	0.58	0.696
TLG	108.23 ± 126.36	219.89 ± 243.05	2.087	0.023

### Survival analysis of the whole group of patients

**Fig. 1:**
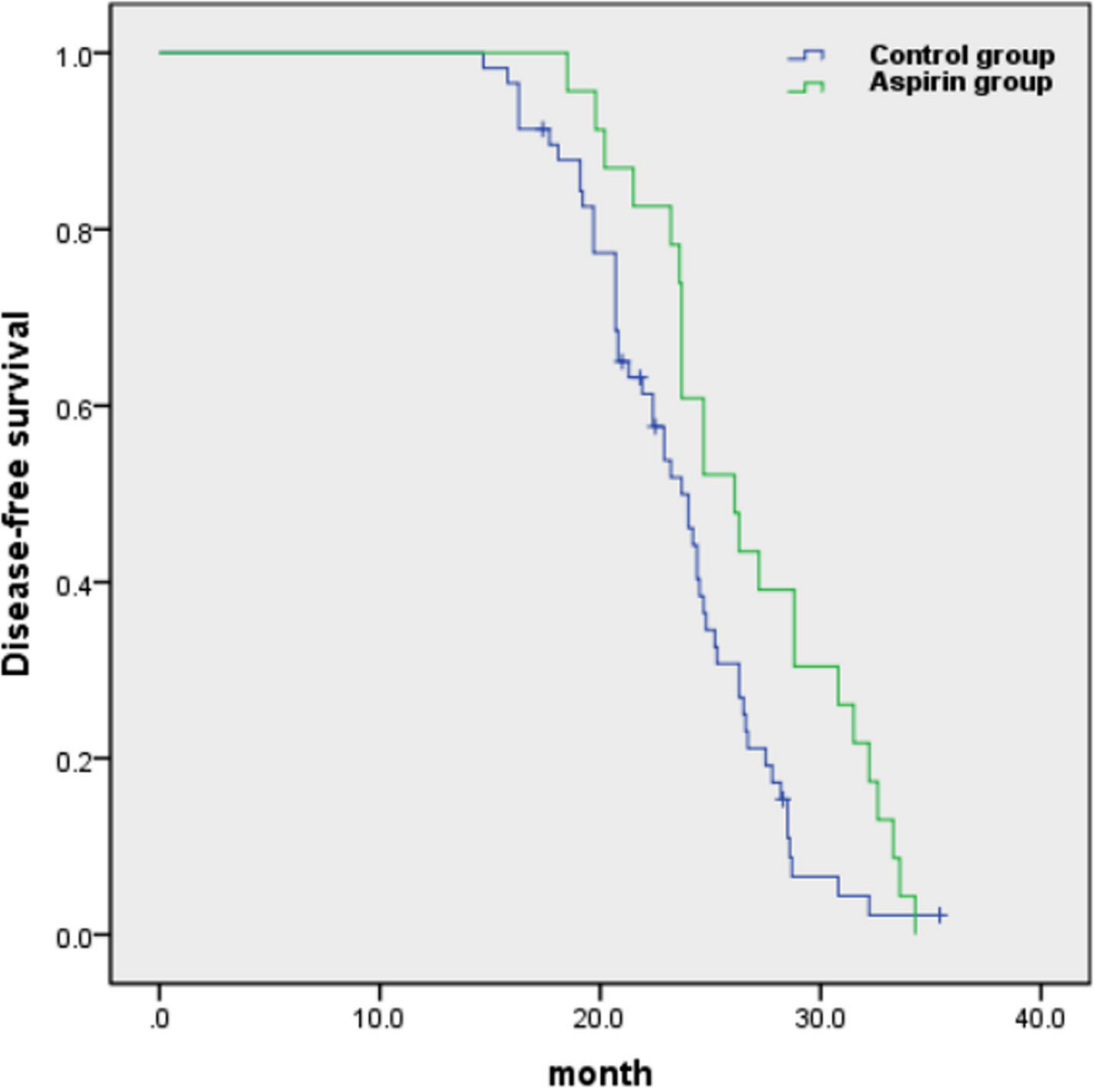
The progression-free survival between aspirin group and control group by Kaplan-Meier curves

The median and range of tumor SUVmax, SUVmean, MTV and TLG were 9.7 (2.9–21.7), 8.5 (2.8–18.6), 15.65 (2.12–110.95) cm^3^ and 110.95 (2.77–1264.83), respectively. The median was the cut off for grouping. The 2-year disease-free survival rates of aspirin group and control group were 56.5 and 41.4%, respectively, suggesting that long-term oral aspirin can improve the disease-free survival of patients with non-small cell lung cancer (Fig. 1).

#### Univariate survival analysis

Kaplan Meier method was used for univariate survival analysis. The factors included: age (≥ 60 years old, < 60 years old), gender, T stage (T1, T2, T3), N stage (N0, N1, N2), pathological type (squamous cell carcinoma, adenocarcinoma and large cell carcinoma), differentiation degree (high differentiation, medium differentiation and low differentiation), aspirin, TNM stage (I, II, III), SUVmax (≥ 9.7, < 9.7)、SUVmean (≥8.5, < 8.5)、MTV (≥15.65, < 15.65)、TLG (≥110.95, < 110.95). The results showed that N stage, TNM stage, aspirin history, SUVmax value, SUVmean and TLG were related to the prognosis of patients (*P* < 0.05). But age, gender, T stage, pathological type, degree of differentiation and MTV had no significant effect on the prognosis (*p* > 0.05). (Table 3).

**Table 3:** Univariable model analysis of disease-free survival

Item	*Chi-square*	*df*	*P* value
Age (years)	0.017	1	0.896
Gender	0.108	1	0.742
T stage	5.717	2	0.056
N stage	24.632	2	0.000
TNM stage	79.590	2	0.000
pathology	0.111	2	0.946
Differentiation	0.117	2	0.943
aspirin	5.240	1	0.022
SUVmax	20.653	1	0.000
SUVmean	7.859	1	0.005
MVT	7.449	1	0.006
TLG	18.296	1	0.000

#### Multivariate survival analysis

Six variables with statistical significance in the above single factor analysis: N stage, TNM stage, aspirin medication history, SUVmax value, SUVmean, TLG were included in the Cox model analysis of multiple factors forward stepwise regression. The results showed that N stage and TNM stage were risk factors affecting prognosis (Table 4).

**Table 4:** Multivariable model analysis of disease-free survival

Item	*B*	*SE*	*Wald*	*df*	*P value*	*95% CI*
N stage	0.556	0.243	5.222	1	0.022	1.082–2.809
TNM stage	1.324	0.364	13.210	1	0.000	1.841–7.678
aspirin	−0.480	0.279	2.961	1	0.085	0.358–1.069
SUVmax	0.745	0.682	1.194	1	0.275	0.554–8.009
SUVmean	0.452	0.666	0.460	1	0.498	0.426–5.799
TLG	−0.255	0.342	0.396	1	0.529	0.412–1.577

## Discussion

In recent years, the role of PET/CT in the early diagnosis, clinical staging and prognosis evaluation of malignant solid tumors has been widely recognized [12, 13, 15]. At present, a widely used tracer for PET/CT is 18F-fluorodeoxyglucose, whose biological behavior is similar to glucose in vivo. The maximum normalized uptake value and the average normalized uptake value reflect the uptake level of 18F-FDG by tumor tissues, and can provide metabolic activity information of tumor cells at the molecular level. It has been reported that SUV, a parameter representing tumor metabolic activity, was a prognostic factor of NSCLC, but the correlation was not as significant as that of stage and tumor volume [10]. The metabolic volume parameters, including tumor metabolic volume (MTV) [16] and total glycolysis (TLG) [17], can represent tumor metabolic load, also have the certain prognostic value. Mazzola et al. proved that 18FDG-PET/CT parameters might be the predictive of response after stereotactic ablative radiotherapy (SABR) for lung metastases [18]. However, whether the metabolic parameters of PET / CT are independent prognostic factors for NSCLC is not consistent at present. Liu J et al. used evidence-based meta-analysis to investigate the prognostic value of PET/CT SUVmax values in patients with operable stage I-II NSCLC [19]. The results showed that the SUVmax value was positively correlated with the risk of recurrence and metastasis. The high SUVmax value indicated that patients were more prone to recurrence and metastasis, and more active treatment measures were needed. Cistaro et al. analyzed 49 patients with stage I-II NSCLC who underwent 18F-FDG PET-CT before surgery and found that SUVmax was an independent prognostic factor [20]. With a SUVmax value of 9 as the cut-off point, the 2-year DFS in the high SUVmax group and the primary tumor size > 3 cm group (37.5%) was significantly lower than the 2-year DFS in the low SUVmax group and the primary tumor size < 3 cm group (90%). Yoo IeR et al. performed a retrospective analysis of 80 patients with T1N0 or T2N0 NSCLC who underwent 18F-FDG PET before surgery. The results showed that SUVmax (*P* = 0.004) and lung adenocarcinoma (*P* = 0.005) were independent prognostic factors for postoperative disease-free survival [21]. Tomita et al. retrospectively analyzed 197 patients with NSCLC who underwent 18F-FDG PET before surgery, suggesting that SUVmax (*P* = 0.0004) and serum CEA levels (*P* < 0.0001) were independent prognostic factors for 5-year survival [22]. Billè et al. studied 413 patients with NSCLC who underwent surgical treatment and survival analysis showed that SUVmax (*P* = 0.006), TNM stage (*P* = 0.0001) and differentiation (*P* = 0.04) were independent prognostic factors affecting survival [23]. All of the above studies have shown that SUVmax was an independent prognostic factor for non-small cell lung cancer. However, there were also some studies showing that SUVmax was not an independent prognostic factor. Downey et al. analyzed 487 patients with NSCLC surgery and found that SUVmax has only independent prognostic value for cTNM staging, but no independent prognostic value for pTNM staging [24]. Hoang et al. studied the prognostic significance of SUVmax in 214 patients with advanced non-small cell lung cancer, and grouped them with a boundary of 11.1. No SUVmax was found to have prognostic value [25]. Therefore, the value of SUVmax in the prognosis of patients with NSCLC remains to be confirmed by further large-scale and prospective studies.

Hypoxia is one of the basic characteristics of solid tumors. Under hypoxic microenvironment, hypoxia-inducible factors in cells are the key transcriptional regulators that mediate adaptive responses in cells [26]. In addition, the expression of Glut-1, which is a key vector of glucose metabolism, is mainly regulated by HIF-1α to meet the energy needs of tumor growth. Molecular biology studies have shown that in the hypoxic environment, Glut-1, which is one of the downstream target genes of HIF-1α, will be up-regulated accordingly, providing tumor tissue with abundant glucose for cell activity [27]. The PET/CT tracer 18F-FDG differs from glucose in that the hydroxyl group at the 2nd carbon atom is substituted by 18, but like glucose, 18F-FDG enters the cell mainly through a transporter such as glucose transporter-1. However, after 18F-FDG enters the cell, it is unable to continue to participate in sugar metabolism through the glycolytic pathway after being catalyzed by hexokinase to produce 6-phosphate-18F-FDG, but accumulated in the cells [28]. Therefore, PET / CT metabolic parameters can reflect the hypoxic state of the tumor to some extent [29]. Our results suggested that the SUVmax, SUVmean and TLG were related to the application of aspirin. There was no significant difference in gender, age, tumor type, degree of differentiation, TNM stage, tumor T stage, and tumor N stage between the aspirin group and the control group. Furthermore, the mean SUVmax of the primary lesions of NSCLC in the aspirin group was 7.3 ± 3.34, which was lower than that of the control group 12.10 ± 5.13. The difference was statistically significant (*P* < 0.05). The SUVmax, SUVmean and TLG value of the aspirin group were lower than that of the control group, suggesting that long-term use of aspirin could reduce the SUVmax value of primary lesions in patients with NSCLC. By performing survival analysis on this group of NSCLC patients, univariate analysis showed that tumor N stage, TNM stage, application of aspirin, and primary SUVmax values, SUVmean and TLG were associated with prognosis. Multivariate analysis suggested that the application of aspirin was not an independent prognostic factor. It only suggested that the tumor N stage, TNM stage were independent prognostic factors. This result was consistent with the findings of SUV in colorectal cancer, and the reason for this result might be related to the small number of cases selected [30]. The 2-year disease-free survival rate of aspirin group was higher than that of the control group, suggesting that long-term oral aspirin can improve the disease-free survival of patients with non-small cell lung cancer. These results suggest that aspirin can improve the prognosis of patients with NSCLC.

Aspirin is a mechanism for affecting the uptake of 18F-FDG by lung cancer cells, reducing the SUVmax value of tumor primary tumors, and thus improving the prognosis of patients. A number of studies have suggested that non-steroidal anti-inflammatory drugs inhibit tumor angiogenesis-related factors [31–33]. Tumor angiogenesis and microenvironmental abnormalities are the result of an imbalance between pro-angiogenic factors and angiogenesis inhibitors in the local microenvironment. In the special environment of tumor tissue, pro-angiogenic factors are significantly increased, and vascular endothelial cells and lymphatic endothelial cells continue to proliferate and migrate under the action of these promoting factors. However, the vascular system thus formed is defective in morphology and function, mainly manifested in the fluctuation of blood flow in spatial distribution and temporal distribution, which leads to the formation of tumor hypoxia microenvironment. In this process, Angiogenesis-related factors play an important role [34]. Aspirin, a COX2 inhibitor, may exert an anti-tumor effect by inhibiting tumor neovascularization and improving the tumor hypoxia microenvironment. Hypoxic microenvironment is the initiator of malignant transformation and even metastasis of tumors, and also plays a central role in tumor chemo-radiation resistance [35, 36]. Therefore, if the tumor microenvironment can be improved, it is expected to reduce the malignant biological potential of tumor cells and improve the efficacy of radiotherapy and chemotherapy, thereby improving the prognosis of patients. Based on the clinical phenomenon that aspirin reduces the uptake of 18F-FDG in NSCLC primary tumors, we speculated that aspirin improved the hypoxia of lung cancer cells, thereby improving the clinical prognosis of patients.

## Conclusions

This article mainly evaluated the effect of aspirin on PET/CT SUVmax in patients with non-small cell lung cancer and its impact on patient prognosis. The results showed that N-stage, TNM stage were independent risk factors for prognosis. Meanwhile, long-term use of aspirin could reduce the PET/CT SUVmax value, SUVmean and TLG of primary non-small cell lung cancer. These provide a new idea for further exploring the role of aspirin in cancer prevention and treatment and further taking timely and effective interventions. Due to the relatively small number of cases in this study, further sample studies are still needed.

## Data Availability

The datasets used and/or analyzed during the current study are available from the corresponding author on reasonable request.

## Abbreviations

18 F-FDG: 18 F-deoxyglucose
SUVmax: Maximum standardized uptake value
PET/CT: Positron emission tomography/computed tomography
NSCLC: Non-small cell lung cancer
Glut: Glucose transporters protein
DFS: Disease-free survival
MTV: Metabolic volume
TLG: The total glycolysis
SUVmean: Mean standardized uptake value
ROI: The region of interest
